# Whole Exome Sequencing in a Series of Patients with a Clinical Diagnosis of Tuberous Sclerosis Not Confirmed by Targeted *TSC1/TSC2* Sequencing

**DOI:** 10.3390/genes12091401

**Published:** 2021-09-10

**Authors:** Erzsebet Kovesdi, Reka Ripszam, Etelka Postyeni, Emese Beatrix Horvath, Anna Kelemen, Beata Fabos, Viktor Farkas, Kinga Hadzsiev, Katalin Sumegi, Lili Magyari, Pilar Guatibonza Moreno, Peter Bauer, Bela Melegh

**Affiliations:** 1 Department of Medical Genetics, Medical School, Szentagothai Research Center, University of Pecs, 7624 Pecs, Hungary; ripszam.reka@pte.hu (R.R.); etelka91@gamma.ttk.pte.hu (E.P.); hadzsiev.kinga@pte.hu (K.H.); sumegi.katalin@pte.hu (K.S.); magyari.lili@pte.hu (L.M.); melegh.bela@pte.hu (B.M.); 2 Molecular Neuroendocrinology Research Group, Institute of Physiology, Center for Neuroscience, Szentagothai Research Center, Medical School, University of Pecs, 7624 Pecs, Hungary; 3 Department of Medical Genetics, Faculty of Medicine, University of Szeged, 6720 Szeged, Hungary; horvath.emese@med.u-szeged.hu; 4 National Institute of Clinical Neurosciences, 1145 Budapest, Hungary; akelemen61@gmail.com; 5 Somogy County Mor Kaposi Teaching Hospital, 7400 Kaposvar, Hungary; fabosbeata@gmail.com; 6 Department of Pediatrics, Faculty of Medicine, Semmelweis University, 1085-Budapest, Hungary; farkas.viktor1@med.semmelweis-univ.hu; 7 Departments of Biochemistry and Medical Chemistry, Medical School, University of Pecs, 7624 Pecs, Hungary; 8 CENTOGENE GmbH, 18055 Rostock, Germany; Peter.Bauer@centogene.com (P.B.); Pilar.Moreno@centogene.com (P.G.M.)

**Keywords:** tuberous sclerosis complex, no mutation identified, Whole Exome Sequencing

## Abstract

Background: Approximately fifteen percent of patients with tuberous sclerosis complex (TSC) phenotype do not have any genetic disease-causing mutations which could be responsible for the development of TSC. The lack of a proper diagnosis significantly affects the quality of life for these patients and their families. Methods: The aim of our study was to use Whole Exome Sequencing (WES) in order to identify the genes responsible for the phenotype of nine patients with clinical signs of TSC, but without confirmed tuberous sclerosis complex 1/ tuberous sclerosis complex 2 (*TSC1/TSC2)* mutations using routine molecular genetic diagnostic tools. Results: We found previously overlooked heterozygous nonsense mutations in *TSC1*, and a heterozygous intronic variant in *TSC2*. In one patient, two heterozygous missense variants were found in polycystic kidney and hepatic disease 1 (*PKHD1)*, confirming polycystic kidney disease type 4. A heterozygous missense mutation in solute carrier family 12 member 5 (*SLC12A5)* was found in one patient, which is linked to cause susceptibility to idiopathic generalized epilepsy type 14. Heterozygous nonsense variant ring finger protein 213 (*RNF213)* was identified in one patient, which is associated with susceptibility to Moyamoya disease type 2. In the remaining three patients WES could not reveal any variants clinically relevant to the described phenotypes. Conclusion: Patients without appropriate diagnosis due to the lack of sensitivity of the currently used routine diagnostic methods can significantly profit from the wider application of next generation sequencing technologies in order to identify genes and variants responsible for their symptoms.

## 1. Introduction

Tuberous sclerosis complex (TSC) is an autosomal dominant, rare disorder with an estimated incidence of 1:6000–1:10,000 and an estimated prevalence of 1:14,000–1:25,000 [1,2]. Until recently the prevalence of TSC was underestimated due to incomplete penetrance and interindividual phenotypic variability [3,4]. The disease affects approximately 2 million people globally [5], and there is no sex or ethnicity predilection [6]. The exact epidemiological data of Hungarian TSC patients is not known, but calculating with the population number in 2021 (9,890,640 citizens), it may affect approximately 900–1600 individuals. 

TSC can affect one or more organ systems leading to a highly variable phenotype [7]. TSC causes tumor or hamartoma formation in the heart, kidney, skin, lung and brain [8]. The most common neurological manifestations of TSC are epilepsy, developmental delay, intellectual disability, behavioral disorders, and autism [9]. 

The first identifiable symptom of TSC is often a cardiac rhabdomyoma (60%), which can be detected as early as the 17th gestational week [10]. Typical dermatological symptoms of TSC are hypomelanotic macules (90%), facial angiofibromas (75%), ungual fibromas (20%), shagreen patches and confetti skin lesions [11]. The dermatological signs can appear at an early age: some patients have hypo-or hyperpigmented skin areas since their birth, while others develop these later in their childhood or in their adolescent years [8]. 

TSC symptoms in the childhood may include symptoms affecting the central nervous system (CNS) as like subependymal nodules (SENs), subependymal giant cell astrocytomas (SEGA), and cortical tuber(s) [12]. In addition, cognitive deficits, autism spectrum disorders and different types of epileptic seizures are very common [8]. Epileptic seizures can occur in 67% of cases early in the first 12 months of their life [13]. TSC associated neuropsychiatric disorders such as autism (40–50%) and attention deficit hyperactivity disorder (ADHD) (30–50%) are manifested in a wide variety and severity in the TSC patient population [14,15]. Renal symptoms occur mainly in young adults, 80% of TSC patients develop angiomyolipomas (AML) or renal cysts (50%) [16]. The treatment of TSC includes pharmacological, surgical, or behavioral interventions. Since this disease is phenotypically highly heterogenic, personalized therapeutic interventions are very important [17].

According to present knowledge TSC is caused by mutations in *TSC1* (tuberous sclerosis complex 1, MIM *605284, 9q34.13) [18] or *TSC2* (tuberous sclerosis complex 2, MIM *191092, 16p13.3) [19] genes, encoding the proteins hamartin (TSC1) and tuberin (TSC2). Similar phenotypes may result from a mutation in either of these two genes, since the encoded proteins bind together to form a functional dimeric complex. In 2012 a third component of the complex was discovered, the TBC1 domain family member 7 (TBC1D7) [20]. TBC1D7 stabilizes TSC the hamartin tuber complex through hamartin [21].

During routine molecular genetic diagnostic testing for *TSC1/TSC2* mutations, 5–25% of the TSC patients do not have any disease-causing mutation in these genes. In the scientific literature these patients are called “no mutation identified” (NMI) TSC cases. They have milder phenotypes compared to patients with *TSC2* mutation, but similar symptoms to those patients with *TSC1* mutation [22,23]. Renal involvement is also common in NMI patients [22,23]. The problem with NMI patients is, that in their case genetic counselors have no explanation regarding the possible genetic and molecular background, which means they cannot give any satisfactory explanations for their symptoms, disease progression and inheritance type.

Next generation sequencing (NGS) has become the core technology for gene discovery in rare disorders. It also has the ability to increase the number of mutations identified in TSC patients [10,24]. Whole Exome Sequencing (WES) technology focuses on the complete coding region of the genome, and scans thousands of genes in the same time and can significantly increase the mutation detection rate.

In this study we analyzed the first cohort of Hungarian TSC NMI patients, in which no *TSC1/TSC2* mutations were identified using Sanger sequencing and multiplex ligation dependent probe amplification (MLPA). The aim of our study was to use WES to find the clinically relevant genes and variants responsible for the described phenotypes of these patients. Based on the results obtained, the patients can get an explanation for their symptoms, an appropriate diagnosis (including inheritance type) and better treatment options. 

## 2. Materials and Methods

### 2.1. The Hungarian TSC Biobank

The TSC biobank at our department is governed by the University of Pecs, as part of the National Biobank Network of Hungary. The governance principles and maintenance management of the Biobank have been approved by the Hungarian National Research Ethics Committee (ETT TUKEB 23476-1/2016/EKU, Budapest, Hungary). During the collection and analysis of DNA samples and processing of the accompanying clinical and personal data the guidelines and regulations of the Helsinki Declaration in 1975 and the currently operative National regulations were followed. Some of the samples come from our department’s genetic counselors, while others come from genetic counselors from various Hungarian health care centers. Patients received genetic counseling in accordance with the human genetic law, including detailed phenotypic analysis and they also gave written consent to genetic testing.

### 2.2. Study Group: TSC NMI Patients

Since 2011, our department in Pecs carries out the molecular genetic analysis of *TSC1* and *TSC2* genes in Hungary. This biobank consists of a total of 204 DNA samples (101 probands and 103 family members). From the 73 DNA proband samples analyzed with Sanger sequencing and MLPA, 9 patients (5 females and 4 males) were NMI cases, the others were either *TSC1* or *TSC2* positive. The 9 NMI DNA samples were further analyzed by WES, to find the possible disease-causing mutation. 

### 2.3. DNA Extraction

Genomic DNA was isolated from peripheral blood samples using E.Z.N.A.® Blood DNA Maxiprep Kit (VWR International Kft., Debrecen, Hungary) according to the manufacturer’s protocol. After extraction, the DNA samples were stored at −80 °C. 

### 2.4. Whole Exome Sequencing (WES)

The DNA samples of the 9 patients were processed at Centogene’s laboratory (Rostock, Germany). The company is College of American Pathologists (CAP) and Clinical Laboratory Improvement Amendments (CLIA) certified, adheres to the “American College of Medical Genetics and Genomics (ACMG) Recommendations for Reporting of Incidental Findings” and does not report on findings which are not directly related to the cause of a disease and not listed in the ACMG guidelines. 

RNA capture baits against approximately 60 Mb of the Human Exome (targeting > 99% of regions in Consensus Coding Sequence (CCDS), RefSeq and Gencode databases) was used to enrich regions of interest from fragmented genomic DNA with Agilent’s SureSelect Human All Exon V6 kit. The generated library was sequenced on an Illumina platform to obtain an average coverage depth of ~100×. Typically, ~97% of the targeted bases are covered >10×. An end to end in-house Centogene bioinformatics pipeline including base calling, alignment of reads to GRCh37/hg19 genome assembly, primary filtering out of low-quality reads and probable artefacts, and subsequent annotation of variants was applied. All disease-causing variants reported in Human Gene Mutation Database (HGMD)®, in ClinVar or in CentoMD® as well as all variants with minor allele frequency (MAF) of less than 1% in genomAD database were considered. Evaluation focused on coding exons along with flanking +/−20 intronic bases. All inheritance patterns were considered. All identified variants were evaluated with respect to their pathogenicity and causality, and these were categorized into classes 1–6. All variants related to the phenotype of the patient, except benign or likely benign variants, were reported. Variants of relevance identified by NGS were continuously and individually in-house (Centogene) validated for quality aspects [25]. All variants reported in the manuscript had quality parameters that were shown to be indicative of a true positive NGS finding; Sanger confirmation was therefore omitted. 

## 3. Results

From the 9 analyzed NMI patients, 6 patients received positive gene finding after WES. Table 1. shows the detailed genotype-phenotype correlations of the nine NMI cases. All new variants were deposited into ClinVar (www.ncbi.nlm.nih.gov/clinvar, accessed on 1 May 2019). under accession numbers: SCV000902239: NM_001256071.2(*RNF213*): c.2875G>T; p.(Gly959*), SCV000899291: NM_000368.4(*TSC1*): c.232G>T; p.(Glu78*), SCV000899290: NM_000548.3(*TSC2*): c.226-6T>G and SCV000902495 NM_001134771.1(*SLC12A5*): c.1417G>A; p.(Val473Ile).

Patient 1 had epilepsy and the Magnetic Resonance Imaging (MRI) showed cortical tubers. WES revealed heterozygous nonsense mutations c.232G>T; p.(Glu78*) in *TSC1*. This variant is classified as likely pathogenic (class 2) and confirmed the genetic diagnosis of tuberous sclerosis type 1.

The second subject (Patient 2) presented renal tumors, angiofibromas on the cheeks, and ungual fibromas on the toes. Similarly to Patient 1, heterozygous nonsense mutation c.1498C>T; p.(Arg500*) was found in *TSC1*, which is classified as pathogenic (class 1). Both variants confirmed the genetic diagnosis of tuberous sclerosis type 1.

Patient 3 had epilepsy, cardiac benign tumor and the MRI results suggested TSC (cortical tuber). A heterozygous intronic variant in *TSC2* with uncertain significance (c.226-6T>G) was identified. It was predicted to disrupt the highly conserved acceptor splice site of exon 4. It is classified as variant of uncertain significance (class 3) leading to the genetic diagnosis of tuberous sclerosis type 2 possible.

In the case of 3 other patients WES revealed disease causing mutations in solute carrier family 12 member 5 (*PKHD1)*, solute carrier family 12 member 5 (*SLC12A5)* and ring finger protein 213 (*RNF213)*. Based on the clinical features of these patients, specific attention was paid during WES to the genes *TSC1* and *TSC2*, but no relevant variants were found. However, pathogenic variants cannot be completely excluded since not all exons were fully covered due to limitations of the method. Patient 4’s clinical signs included polycystic kidney, epilepsy and the MRI showed numerous typical signs of TSC (cortical tubers). Two heterozygous missense variants: c.5513A>G; p.(Tyr1838Cys) and c.3747T>G; p.(Cys1249Trp) were found in *PKHD1*. Both variants are classified as pathogenic (class 1) confirming polycystic kidney disease type 4. Since the *PKHD1* mutations can only partially explain the phenotype of the patient, Whole Genome Sequencing (WGS) was considered to uncover genetic variants not covered by WES.

Patient 5 presented hypomelanotic macule, epileptic seizures and white matter lesions as clinical features. Specific attention was paid during WES to early infantile epileptic encephalopathy (EIEE) related genes (*AARS, ALG13, ARHGEF9, ARV1, ARX, CACNA1A, CDKL5, DNM1, DOCK7, EEF1A2, FRRS1L, GABRA1, GABRB3, GNAO1, GRIN2B, GUF1, HCN1, ITPA, KCNA2, KCNB1, KCNQ2, KCNT1, NECAP1, PCDH19, PIGA, PLCB1, PNKP, SCN1A, SCN2A, SCN8A, SCN9A, SLC12A5, SLC13A5, SLC1A2, SLC25A12, SLC25A22, SLC35A2, SPTAN1, ST3GAL3, STXBP1, SZT2, TBC1D24, WWOX, AP3B2, KCNT2, HNRNPU, CAD, UBA5, FGF12, GABRB1, MDH2, YWHAG, DENND5A, SCN1B, GRIN2D, SYNJ1, SIK1*), to *TSC1* and *TSC2* and to *IKBKG*-related disorder. WES analysis could not detect any relevant variant in these genes except in *SLC12A5*, where a heterozygous missense mutation c.1417G>A; p.(Val473Ile) with uncertain significance (class 3) was found. The variant is linked to cause susceptibility to idiopathic generalized epilepsy type 14 (EIG14). 

Patient 6 showed epilepsy (epileptic seizures but the exact starting point could not be determined) and small hypopigmented spots. The MRI results suggested the possibility of TSC (cortical lesions). A heterozygous nonsense variant c.2875G>T; p.(Gly959*) in *RNF213* was identified. The variant is classified as likely pathogenic (class 2). Pathogenic variants in the *RNF213* have been associated with susceptibility to Moyamoya disease type 2.

In the remaining 3 patients WES could not reveal any variants clinically relevant to the described phenotypes of these patients. It is important to mention, that pathogenic variants at these three patients cannot be completely excluded, since not all exons were fully covered due to limitations of the method. 

In Patient 7, who had renal angiomyolipoma which led to right kidney nephrectomy; multiplex angiomyolipomatosis in the left kidney, adenoma sebaceum on the cheeks and fibromas on the toes. During the analysis of the sample, specific attention was paid during WES to the genes associated with multiple endocrine neoplasia (*CDKN1B, RET, MEN1*) and TSC genes, but no variants clinically relevant to the described phenotype of the patient were observed. 

The psychomotor development of Patient 8 was slow, MRI showed reduction in the volume of paraventricular white matter and right-side dominant polymicrogyria at the area of aqueductus cerebri. Front part of both temporal lobes and the frontal lobes were affected by polymicrogyria with moderate severity. He also had vermis hypoplasia. As dermatological signs halo nevus, one leaf-shaped hypomelanotic macule on the torso, one palm size at the right knee bend were observable. In Patient 8 based on the clinical information specific attention was paid to the genes in neuronal migration disorders panel (*ACTB, ACTG1, ARFGEF2, ARX, COL18A1, COL4A1, CPT2, DCX, EMX2, EOMES, FGFR3, FH, FKRP, FKTN, FLNA, ADGRG1, IER3IP1, ISPD, LAMA2, LAMC3, LARGE, MED12, MEF2C, OCLN, PAFAH1B1, PAX6, PEX7, POMGNT1, POMT1, POMT2, PQBP1, RAB18, RAB3GAP1, RAB3GAP2, RELN, SNAP29, SRPX2, TUBA1A, TUBA8, TUBB2B, TUBB3, VDAC1, WDR62*) and on lissencephaly and brain malformation panel (*ACTB, ACTG1, ADGRG1, ARX, CDK5, COL6A1, COL6A2, COL6A3, DCX, DYNC1H1, EOMES, FKRP, FKTN, ISPD, KATNB1, KIF2A, KIF5C, LAMA2, LAMB1, LARGE, LMNA, NDE1, PAFAH1B1, POMGNT1, POMT1, POMT2, RELN, SELENON, TUBA1A, TUBB, TUBB2A, TUBB2B, TUBB3, TUBG1, VLDLR, WDR62, YWHAE*), but no relevant variants in these genes was found. WGS is considered which can add an additional 15–18% clarification rate compared to WES.

The MRI of Patient 9 showed cortical tuber, SENs detected at the right side of the frontal ventricle and a cyst was detected at the area of the corpus pineale. Special attention was paid during WES to the *TSC1/TSC2*, but no variants clinically relevant to the described phenotype of the patient was detected.

## 4. Discussion

The goal of our very first TSC NMI study was to identify with a next generation sequencing tool (WES) the possible genes and variants which can be responsible for the clinical signs of our 9 Hungarian TSC NMI patients. With WES we found in 6 cases the genes responsible for their symptoms: previously overlooked *TSC1/TSC2* mutations and disease-causing variants in other genes like *PKHD1, SLC12A5* and *RNF213*. However, in 3 patients WES could not reveal any variants clinically relevant to the described phenotypes of these patients.

In the recent years several research studies were published, in which different NGS technologies were used with more or less success to increase the detection rate of *TSC1/TSC2* mutations in TSC NMI patients. The first study which used NGS technology to find previously missed *TSC1* and *TSC2* mutations in 38 NMI subjects was performed by Qin and coworkers in 2010 [26]. With Roche 454 Sequencing on Genome Sequencer FLX system they found five heterozygous mutations in *TSC1/TSC2*, and only 2 patients had mosaic mutations. The used technique was not able to detect mutations in promoter, introns and regulatory sequences. In another article, using HaloPlex targeted capture NGS technology, from the analyzed 7 NMI patients, pathogenic mutations were revealed in 3 cases, while the others had variants of uncertain significance (VUS) [10]. A third paper using Illumina HiSeq 2000 and 2500 platforms [27] analyzed not only the *TSC1/TSC2* genes with 10 kb of upstream and downstream sequences, but other genes which could be potential candidates for a possible *TSC3* gene (*DEPTOR, PRAS40, TBC1D7, DEPDC5, NPRL2,* and *NPRL3*), because of their role in mechanistic target of rapamycin (mTOR) signaling pathway. However, they could not find any pathogenic variants in the above mentioned six genes. In 2018, a Russian study performed WES as a part of their experiments on five NMI patients, using Illumina Nextera Kit for exome enrichment and Illumina MiSeq, but they failed to identify disease-causing mutation candidate genes among their NMI patients [28].

A recent study with Chinese TSC patients analyzed genomic variants in the non-*TSC1/TSC2* genes participating in the mTOR pathway and found that 12 mTOR pathway related genes in 11 of their 40 NMI patients. Ten genes were at the upstream of mTOR Complex 1 (*mTORC1)* (*WNT5B, FZD4, FZD6, FZD9, GSK3B, MAP2K2, IRS1, PIK3CA, PIK3R2* and *CHUK*), while two genes (*LPIN1* and *PRKCG*) at the downstream of *mTORC1* and mTOR Complex 2 *(mTORC2)*. The 12 variants they found were classified as VUS according to the ACMG guidelines due to the lack of direct evidence to support their involvement in TSC. They could not test these variants for mosaic or intronic mutations due to the unavailability of the tissue samples and relatively low sequencing depth (100–200×). The authors concluded, that “disease-causative genes” of TSC other than *TSC1/TSC2* may exist [29].

In this study we analyzed 9 Hungarian TSC NMI patient with WES. In the case of 3 patients we found that they have previously overlooked *TSC1/TSC2* mutations (Patient 1–3). In the cases of Patient 1 and 2 WES analysis revealed pathogenic, resp. likely pathogenic variants. Based on the clinical sign and WES of these patients, the diagnosis of TSC was verified. Both *TSC1* variants create premature stop codon. The c.232G>T; p.(Glu78*) variant is formerly unpublished based on the online available TSC mutation databases, while the variant c.1498C>T; p.(Arg500*) has been previously described as disease causing for TSC by van Slegtenhorst and coworkers [30]. In one patient a heterozygous intronic variant in *TSC2* with uncertain significance c.226-6T>G was identified. This variant is predicted to disrupt the highly conserved acceptor splice site of exon 4. Potential effect on splicing is routinely analyzed at Centogene using several in silico prediction programs. For the c.226-6T>G variant in *TSC2*, these programs predicted a negative/detrimental effect on the splice acceptor. “Splice Site Finder” assigned a splice acceptor score of 74.93 to the wild-type sequence (max value =100.00), while not at all scoring the mutant sequence.

In the case of 3 other patients WES revealed disease causing mutations in *PKHD1*, *SLC12A5* and *RNF213*. Two heterozygous missense variants were found in *PKHD1* (c.5513A>G; p.(Tyr1838Cys) and c.3747T>G; p.(Cys1249Trp)) in Patient 4. Both variants found in this gene were previously described as disease causing mutations for polycystic kidney disease [31,32]. Pathogenic *PKHD1* variants are causative for polycystic kidney disease type 4 with or without hepatic disease, also known as autosomal recessive polycystic kidney and hepatic disease (ARPKD). Most of the cases are diagnosed at birth or late in pregnancy, but there are some cases where they were identified in older people with moderately severe symptoms [33,34]. The majority of individuals with ARPKD presented in the neonatal period show “potter” phenotype: massively enlarged kidneys, pulmonary hypoplasia, characteristic facies, and contracted limbs with club feet. The renal disease is characterized by nephromegaly, hypertension, and varying degrees of renal dysfunction [34,35]. The ultrasound picture of the kidneys usually shows bilaterally enlarged hyperechoic kidneys with poor corticomedullary differentiation, retained reniform contour, and multiple tiny cysts confined to distal tubules and collecting ducts. The size and place of the cysts can vary and often accompanied by some degree of interstitial fibrosis [35].

The establishment of genotype–phenotype correlations for *PKHD1* is not simple [36], and there are some phenotypic overlaps between ARPKD other diseases, among this TSC, which suggests functional relationship between the causative genes/proteins and the signaling pathway, in this case the mTOR [37]. Polycystin-1 and the TSC1/TSC2 tumor suppressor complex both act to suppress the activity of mTOR resulting in apoptosis [38]. In our case, the common symptom between ARPKD and TSC was the renal cyst, however, *PKHD1* mutations can only partially explain the phenotype of the patient. Based on the WES results we do not have the genetic explanation neither for the epilepsy nor the cortical tubers. During the analysis specific attention was paid to the *TSC1/TSC2* genes, but no relevant variant in these genes were detectable. However, pathogenic variants cannot be completely excluded since not all exons were fully covered due to limitations of the method. WGS is more powerful tool compared to detect known genetic variants, small insertions/deletions and Copy Number Variations (CNVs) within the regions of the genome covered by WES. Given the fact that *PKHD1* mutations can only partially explain the phenotype in the patient, we think WGS should be considered to uncover genetic variants not covered by WES. WGS typically results in ~10–20% additional genetic diagnoses.

In Patient 5 WES analysis could not detect any relevant variant in these genes except in *SLC12A5*, which is linked to cause susceptibility to idiopathic generalized epilepsy type 14 (EIG14). However, other pathogenic variants cannot be completely excluded since not all exons were fully covered due to limitations of the method. There are only two publications in the literature, which deal with EIG14. Kahle et al. [39] reported 8 patients with French Canadian origin with idiopathic generalized epilepsy with onset between 3–21 years of age. Seizure types were generalized tonic-clonic, absence, and myoclonic. EEG showed generalized spike-wave discharges or diffuse theta waves. None of the patients had febrile seizures. The transmission pattern of EIG14 in the families reported was consistent with autosomal dominant inheritance and incomplete penetrance. In the second manuscript—published by Puskarjov and coworkers [40], a heterozygous p.Arg952His variant in *SLC12A5* was found in three affected patients of an Australian family with febrile seizures. The authors suggested that the decrease in *SLC12A5*-dependent hyperpolarizing inhibition would promote triggering of seizures. The decreased dendritic spine formation could lead to desynchronization of overall excitability which contribute to the formation of seizures. 

Interestingly, a known heterozygous nonsense variant on *RNF213* (c.2875G>T; p.(Gly959*)) was identified in Patient 6, leading to premature stop codon. Pathogenic variants in the *RNF213* gene are linked to Moyamoya disease, which is probably multifactorial and polygenic disease with various clinical presentations and inheritance type. It is characterized by bilateral internal carotid artery stenosis and abnormal collateral vessels. The abnormal vessels look like a “puff of smoke” on cerebral angiogram. Affected individuals can develop transient ischemic attacks, or cerebral infarction. The rupture of the collateral vessels can lead to intracranial hemorrhage [41,42]. The *RNF213* was the first described susceptibility gene for the disease [43,44], however the exact mechanism how the RNF213 abnormality relates to the disease is still unknown. Based on the literature *RNF213* is an important susceptibility gene of among the East Asian population, but it is rare in Western countries [45,46]. We did a search the scientific literature (Pubmed), most of the articles deal with the polymorphism p.Arg4810Lys, the founder variant in East Asian (Japanese, Korean, and Chinese) patients [47,48]. A GWAS conducted among 38 European patients failed to identify any major founder variant associated with Moyamoya disease [49]. Hever’s study [50] suggest that there is a distinct Western Moyamoya disease phenotype, characterized by a more pronounced female prevalence, later disease onset than the average, relative lack of family history and decreased possibility of hemorrhagic stroke compared to Asian study groups [50]. Based on the clinical features of Patient 6, specific attention was paid during WES to the genes *TSC1* and *TSC2*, but no relevant variants in these genes were found. However, pathogenic variants cannot be completely excluded since not all exons were fully covered due to limitations of the method. Similarly to Patient 4, WGS was considered to uncover genetic variants not covered by WES to find genetic variants to explain the remaining symptoms.

In the remaining 3 patients WES could not reveal any variants clinically relevant to the described phenotypes of these patients. 

Based on the literature there more options to explain the genetic status of NMI patients:

(1) Mutation detection failure during routine diagnostic. This was the case in 3 of our NMI patients (Patients 1–3). (2) Mosaicism can also be an explanation based on the literature (example: [27], so we considered mosaicism as a possible explanation for the genetic status of our TSC NMI patients. However, the WES analysis by Centogene did not confirmed mosaicism in our subjects. (3) Mutations in introns affecting splicing, not near exons, promoter and enhancer regions which are not covered during routine molecular diagnostic testing. In one of our cases (Patient 3), intronic mutation was found. (4) Possibility of a currently unknown TSC gene. According to our knowledge, there are no publications which confirms the presence of a new component of TSC which is responsible for the disease. The routine pipeline of Centogene uses >25% allele frequency to detect mosaic variants.

There are several limitations in our study, therefore our results should be interpreted with caution: (1) The number of patients we have analyzed is not large. (2) The WES results were interpreted in the context of clinical findings and laboratory data. (3) Only variations in genes potentially related to the proband’s medical condition were reported. Specific genetic events like CNVs, translocations and repeat expansions may not be reliably detected with Exome Sequencing. In addition, due to limitations in technology, certain regions may either not be covered or may be poorly covered, where variants cannot be confidently detected. It also cannot detect large deletions/duplications. (4) It might have been advantageous to proceed the analysis with other approaches (WGS, long-read sequencings, 3rd generation sequencing), unfortunately as these are not part of the diagnostic panel yet, the patients did not agree to such a proceed.

## 5. Conclusions

Patients without the appropriate diagnosis due to the lack of sensitivity of the currently used routine diagnostic methods, or patients with unclear phenotype can significantly profit form the wider application of next generation sequencing technologies to find the genes and variants responsible for their symptoms to improve their treatment options and thereby their life quality. In agreement with other researcher groups [10,27], we think that with the use of NGS technologies during routine genetic diagnostics the number of NMI patients will be reduced. 

## Figures and Tables

**Table 1:** Genotype-phenotype features of TSC NMI patients.

	Patient No.	P1	P2	P3	P4		P5	P6	P7	P8	P9
	**Gender (M/F)**	M	F	M	F		M	F	F	M	M
	**Age (Y)**	25	36	8	13		13	8	52	11	4
	**TSC diagnostic** **status**	*Possible*	*Definite*	*Definite*	*Possible*		*Possible*	*Possible*	Definite	Possible	Definite
**Genetics**	**Affected gene**	*TSC1*	*TSC1*	*TSC2*	*PKHD1*		*SLC12A5*	*RNF213*	NA	NA	NA
	**c.DNA change**	c.232G > T	c.1498C > T	c.226-6T > G	c.3747T > G	c.5513A > G	c.1417G > A	c.2875G > T			
	**Protein change**	p.Glu78X	p.Arg500X	N/A	p.Cys1249Trp	p.Tyr1838Cys	p.Val473Ile	p.Gly959X			
	**Variant type**	Nonsense	Nonsense	Splicing	Missense	Missense	Missense	Nonsense			
	**Zygosity**	Heterozygous	Heterozygous	Heterozygous	Heterozygous	Heterozygous	Heterozygous	Heterozygous			
	**Classification**	Likely pathogenic (2)	Pathogenic (1)	VUS (3)	Pathogenic (1)	Pathogenic (1)	VUS (3)	Likely pathogenic (2)			
	**Diagnosis after WES**	TSC type 1	TSC type 1	TSC type 2	Polycystic kidney disease type 4 with or without hepatic disease		Idiopathic generalized epilepsy type 14	Moyamoya disease type 2			
	**Inheritance**	AD	AD	AD	AR		AD	AD			
**Skin**	**Hypomelanotic macule**	−	−	−	−		**+**	**+**	−	**+**	−
	**Adenoma sebaceum**	−	**+**	−	−		−	−	**+**	−	−
	**Ungual fibroma**	−	**+**	−	−		−	−	**+**	−	−
	**Shagreen patch**	−	−	−	−		−	−	−	−	−
**Central nervous system**	**SEN**	−	−	−	−		−	−	−	−	**+**
	**SEGA**	−	−	−	−		−	−	−	−	−
	**Cortical tuber**	**+**	−	**+**	**+**		−	−	−	−	**+**
	**Epilepsy**	**+**	−	**+**	**+**		**+**	**+**	−	−	−
	**Other**	−	−	−	−		White matter lesions	Cortical lesions	−	Slow psychomotor development, Vermis hypoplasia, Polymicrogyria	Corpus pineale cyst
**Kidney**	**Renal cyst**	−	−	−	**+**		−	−	−	−	−
	**Renal tumor**	−	**+**	−	−		−	−	−	−	−
	**Angiomyolipoma**	−	−	−	−		−	−	**+**	−	−
**Heart**	**Ventricular septal defect**	−	−	−	−		−	**+**	−	−	−
	**Rhabdomyoma**	−	−	**+**	−		−	−	−	−	−
**Other symptoms**		−	−	−	−		−	−	Lymphangioleiomyomatosis	Right hallux onychodystrophy	−

Abbreviations: *TSC1*: tuberous sclerosis 1, *TSC2:* tuberous sclerosis 2, *PKHD1:* fibrocystin, *SLC12A5:* solute carrier family 12 member 5, *RNF213:* ring finger protein 213, AD: autosomal dominant, AR: autosomal recessive, WES: whole exome sequencing, SEN: subependymal nodule, SEGA: subependymal glial cell astrocytome, VUS: variant of uncertain significance.

## Data Availability

All data are presented in the manuscript.
